# Bactericidal and Sterilizing Activity of a Novel Regimen with Bedaquiline, Pretomanid, Moxifloxacin, and Pyrazinamide in a Murine Model of Tuberculosis

**DOI:** 10.1128/AAC.00913-17

**Published:** 2017-08-24

**Authors:** Si-Yang Li, Rokeya Tasneen, Sandeep Tyagi, Heena Soni, Paul J. Converse, Khisimuzi Mdluli, Eric L. Nuermberger

**Affiliations:** a Center for Tuberculosis Research, Department of Medicine, Johns Hopkins University, Baltimore, Maryland, USA; b Global Alliance for Tuberculosis Drug Development, New York, New York, USA

**Keywords:** Mycobacterium tuberculosis, bedaquliine, mouse, moxifloxacin, pretomanid, pyrazinamide

## Abstract

New regimens based on 2 or more novel agents are sought to shorten or to simplify treatment of tuberculosis (TB), including drug-resistant forms. Prior studies showed that the novel combinations of bedaquiline (BDQ) plus pretomanid (PMD) plus pyrazinamide (PZA) and PMD plus moxifloxacin (MXF) plus PZA shortened the treatment duration necessary to prevent relapse by 2 to 3 months and 1 to 2 months, respectively, compared with the current first-line regimen, in a murine TB model. These 3-drug combinations are now being studied in clinical trials. Here, the 4-drug combination of BDQ+PMD+MXF+PZA was compared to its 3-drug component regimens and different treatment durations of PZA and MXF were explored, to identify the optimal regimens and treatment times and to estimate the likelihood of success against drug-resistant strains. BDQ+PMD+MXF+PZA rendered all mice relapse-free after 2 months of treatment. PZA administration could be discontinued after the first month of treatment without worsening outcomes, whereas the absence of MXF, PZA, or BDQ administration from the beginning necessitated approximately 0.5, 1, or 2 months, respectively, of additional treatment to attain the same outcome.

## INTRODUCTION

The current estimate of the global burden of tuberculosis (TB) was recently revised upward by the World Health Organization to 10.4 million incident cases for 2015, reflecting new information obtained from countries in which the disease is highly endemic. Nearly 600,000 new cases of multidrug-resistant (MDR) TB occur annually (1). Current recommendations call for up to 2 years of treatment with second-line drugs, which are poorly tolerated, toxic, more difficult to administer, and less effective than the 6-month short-course regimen consisting of rifampin (RIF), isoniazid (INH), pyrazinamide (PZA), and ethambutol (EMB), for drug-susceptible (DS) TB. Regimens containing at least 6 drugs, including newer fluoroquinolones in high doses, an injectable agent, clofazimine, PZA, and high-dose INH, have shown potential as effective 9-month regimens in MDR-TB cases with minimal bacillary resistance to second-line drugs (2–4). However, these regimens remain quite cumbersome to administer and are not expected to be as effective in the setting of resistance to fluoroquinolones and/or injectable agents (3). Novel regimens based on two or more oral agents with little or no preexisting resistance would provide simpler, more universally active regimens. If such novel regimens are more effective than the current first-line regimen for drug-susceptible TB, then they may shorten and simplify treatment for pulmonary TB irrespective of resistance to existing drugs.

Pretomanid (PMD) plus moxifloxacin (MXF) plus PZA showed bactericidal and sterilizing activities that were at least as effective as those of RIF+INH+PZA in a murine model of TB (5), shortening the treatment period necessary to prevent relapse by at least 1 month, compared to RIF+INH+PZA+EMB. In terms of bactericidal activity, the findings translated well to TB patients in a 14-day early bactericidal activity (EBA) trial (ClinicalTrials registration no. NCT01215851) (6), as well as an 8-week phase 2b trial (NC-002 trial; ClinicalTrials registration no. NCT01498419) (7) in which PMD+MXF+PZA shortened the time to sputum culture conversion in DS-TB patients and performed at least as well in MDR-TB patients as the first-line regimen performed in control DS-TB patients. These results prompted the phase 3 STAND trial (ClinicalTrials registration no. NCT02342886) to evaluate the potential of the PMD+MXF+PZA regimen to reduce the treatment duration for DS-TB and MDR-TB to 4 and 6 months, respectively.

Similarly, bedaquiline (BDQ) plus PMD plus PZA was shown to be more efficacious than RIF+INH+PZA in the same murine model, with results suggesting the potential to shorten treatment duration by 2 to 3 months (8). In a 14-day EBA trial (9), BDQ+PMD+PZA had activity comparable to or greater than that of RIF+INH+PZA+EMB. An 8-week phase 2b trial (NC-005 trial; ClinicalTrials registration no. NCT02193776) evaluating this regimen in DS-TB is ongoing.

Here, the bactericidal and sterilizing activities of a regimen merging the PMD+MXF+PZA and BDQ+PMD+PZA regimens, i.e., BDQ+PMD+MXF+PZA, were evaluated alongside regimens lacking BDQ, MXF, or PZA, in the same murine model. The impact of shorter durations of PZA or MXF+PZA treatment in the BDQ+PMD+PZA and BDQ+PMD+MXF+PZA regimens was also explored. The results indicate that the 4-drug BDQ+PMD+MXF+PZA regimen has greater bactericidal and sterilizing activities than either the BDQ+PMD+PZA regimen or the PMD+MXF+PZA regimen and deserves greater priority for evaluation in clinical trials as a treatment-shortening regimen for DS-TB and MDR-TB. Although both PZA and MXF contribute treatment-shortening activity to the 4-drug regimen, the loss of either component alone (e.g., due to resistance) results in a regimen that is still superior to the current first-line regimen. Moreover, the efficacy of BDQ+PMD alone is still comparable to that of the first-line regimen. This finding suggests that identification of resistance to PZA and MXF could prompt their replacement with additional agents and still result in a novel regimen that is at least as effective as first-line therapy.

## RESULTS

### Lung CFU counts during treatment in experiment 1.

This experiment compared the efficacy of the 4-drug regimen of BDQ+PMD+MXF+PZA to that of BDQ+PMD+MXF, BDQ+PMD+PZA, and PMD+MXF+PZA, with the aim of measuring the contributions of PZA, MXF, and BDQ to the 4-drug regimen. Two doses of PMD were studied in the PMD+MXF+PZA regimen in order to model the two PMD doses, 100 mg and 200 mg, studied in the NC-002 and STAND trials. CFU burdens were assessed for all regimens after the first month of treatment and for the RIF+INH+PZA, PMD_50_+MXF+PZA, PMD_100_+MXF+PZA, and BDQ+PMD+MXF regimens after 2 and 3 months of treatment.

After 1 month of treatment, an ∼3.25-log_10_ reduction of CFU was observed in the RIF+INH+PZA group, with incrementally greater reductions in the PMD_50_+MXF+PZA, BDQ+PMD+MXF, and PMD_100_+MXF+PZA groups (Table 1), but mice treated with BDQ+PMD+PZA or BDQ+PMD+PZA-MXF experienced an additional 2-log_10_ reduction, with no increased killing observed with the addition of MXF. At month 1, all regimens except for the low-dose PMD regimen were significantly (*P* < 0.0001) more active than the RIF+INH+PZA standard; at that time point, BDQ+PMD+PZA-MXF was significantly (*P* < 0.0001) more active than all regimens except for BDQ+PMD+PZA. The decreasing of the CFU burden continued over the next month at a rate greater than that of RIF+INH+PZA, although only PMD+MXF+PZA was significantly (*P* = 0.005) more active. By month 3, both PMD+MXF+PZA regimens and the BDQ+PMD+MXF regimen showed significantly greater killing activity (*P* < 0.0001), with the latter regimen rendering all mice culture negative.

**TABLE 1:** Lung CFU counts assessed during treatment and proportions of mice relapsing after treatment completion in experiment 1

Drug regimen	Log_10_ CFU count (mean ± SD)				No. relapsing/total no. (%)				
	Day 0	Month 1	Month 2	Month 3	Month 1.5	Month 2	Month 3	Month 4	Month 5
None	7.42 ± 0.19								
RIF+INH+PZA		4.16 ± 0.24	2.47 ± 0.26	1.42 ± 0.12				10/15 (67)	2/15 (13)
PMD_50_+MXF+PZA		3.97 ± 0.20	1.84 ± 0.56	0.28 ± 0.38				6/14 (43)	0/14 (0)
PMD_100_+MXF+PZA		3.37 ± 0.19	1.39 ± 0.54	0.22 ± 0.32			10/14 (71)	3/15 (20)	
BDQ+PMD_100_+MXF		3.61 ± 0.15	2.33 ± 0.19	0.00 ± 0.00			2/15 (13)	0/14 (0)	
BDQ+PMD_100_+PZA		1.71 ± 0.11			13/14 (93)	0/15 (0)	0/15 (0)		
BDQ+PMD_100_+MXF+PZA		1.74 ± 0.28			3/15 (20)	0/15 (0)	0/15 (0)		

At day 0, all mice harbored PMD-resistant CFU. The mean ± standard deviation (SD) log_10_ CFU count on plates containing PMD at 2 μg/ml was 2.93 ± 1.30 CFU. At month 1, PMD-resistant CFU were recovered from 2 of 5 mice receiving RIF+INH+PZA (mean CFU count, 0.64 ± 0.90 CFU [*n* = 5]) and 4 of 5 mice in each PMD+MXF+PZA group (mean CFU count, 1.34 ± 0.81 CFU [*n* = 15]) (*P* = 0.003 by *t* test). PMD resistance could not be assessed in mice during treatment with BDQ-containing regimens, because the charcoal used to prevent BDQ carryover also bound PMD and prevented its selective effect. At month 2, PMD-resistant CFU were recovered from only 1 mouse receiving PMD_50_+MXF+PZA, but they represented 35% of the total CFU in that mouse. No PMD-resistant CFU were recovered at month 3.

At day 0, the mean ± SD log_10_ CFU count on plates containing BDQ at 0.06 or 0.125 μg/ml was 2.73 ± 0.32 CFU. At month 1, CFU were recovered on BDQ-containing plates from 2 of 5 mice receiving RIF+INH+PZA (mean CFU count, 0.46 ± 0.64 CFU [*n* = 5]), 7 of 15 mice receiving any PMD+MXF+PZA regimen (mean CFU count, 0.35 ± 0.52 CFU [*n* = 15]), and 0 of 15 mice in BDQ-containing groups (mean CFU count, 0.34 ± 0.81 CFU [*n* = 15]) (*P* < 0.01 for BDQ-containing regimens versus all other regimens, compared in the aggregate). No CFU were recovered on BDQ-containing plates at month 2 or month 3.

### Relapse after treatment completion in experiment 1.

Relapse was assessed in select groups after 1.5, 2, 3, 4, and 5 months of treatment, and mice were sacrificed after a further 3 months without treatment (Table 1). Although the CFU burdens at month 1 for mice treated with BDQ+PMD+PZA or BDQ+PMD+PZA-MXF were essentially identical, at month 1.5 there were significantly (*P* = 0.0001) fewer relapsing mice in the BDQ+PMD+MXF+PZA group. After 2 or 3 months of treatment, both of these groups were relapse free. At the month 3(+3) relapse time point, >70% of mice treated with PMD+MXF+PZA without BDQ relapsed, whereas only 13% of mice treated with BDQ+PMD+MXF without PZA relapsed (*P* = 0.0025), confirming the greater contribution of BDQ, compared to PZA, to the sterilizing activity of the 4-drug regimen. After 4 months of treatment, the latter group had no relapses, but 20% of mice in the PMD+MXF+PZA group relapsed (Table 1). Reducing the PMD dose by one-half resulted in twice as many mice relapsing at this time point, although the difference was not statistically significant. Fig. S1 in the supplemental material shows the decreases in the numbers of relapsing mice and the reductions in CFU burdens in the different treatment groups.

PMD-resistant CFU were found for 3 of 10 mice that relapsed after 3 months of treatment with PMD_100_+MXF+PZA, representing 1 to 10%, 38%, and 100% of the total CFU. No PMD-resistant CFU were found for the 2 mice that relapsed after 3 months of treatment with BDQ+PMD+MXF. A single PMD-resistant colony was found for 1 of 6 mice that relapsed after 4 months of treatment with PMD_50_+MXF+PZA, representing approximately 0.2% of the total CFU count. No PMD-resistant CFU were found among mice that relapsed after treatment with PMD_100_+MXF+PZA or RIF+INH+PZA for 4 months or mice that relapsed after 5 months of treatment with RIF+INH+PZA.

### Lung CFU counts during treatment in experiment 2.

This experiment again compared the efficacy of the 4-drug regimen of BDQ+PMD+MXF+PZA to that of BDQ+PMD+MXF and BDQ+PMD+PZA, to confirm the contributions of PZA and MXF to the 4-drug regimen, and it also assessed the most appropriate treatment duration for MXF and PZA in the BDQ+PMD+MXF+PZA combination, by including regimens in which PZA or MXF+PZA treatment was discontinued after the first month. The 2-drug BDQ+PMD combination was included as an additional control. Over the first month of treatment, the bactericidal activities of the regimens were ranked as follows: BDQ+PMD < RIF+INH+PZA < BDQ+PMD+MXF ≪ BDQ+PMD+PZA < BDQ+PMD+MXF+PZA. Only BDQ+PMD+PZA and BDQ+PMD+MXF+PZA were significantly more active than RIF+INH+PZA (Table 2). At month 2 and month 3, BDQ+PMD and RIF+INH+PZA had similar activities, while the BDQ+PMD+MXF regimen was significantly more active (*P* < 0.0001) and rendered 3 of 5 mice culture negative at month 3.

**TABLE 2:** Lung CFU counts assessed during treatment and proportions of mice relapsing after treatment completion in experiment 2

Drug regimen^*a*^	Log_10_ CFU count (mean ± SD)				No. relapsing/total no. (%)				
	Day 0	Month 1	Month 2	Month 3	Month 1	Month 1.5	Month 2	Month 3	Month 4
None	8.09 ± 0.24								
RIF+INH+PZA		4.93 ± 0.22	2.97 ± 0.07	1.05 ± 0.28					5/15 (33)
BDQ+PMD		5.33 ± 0.26	2.78 ± 0.10	1.39 ± 0.45					1/15 (7)
BDQ+PMD+MXF+PZA		1.96 ± 0.25			15/15 (100)	4/17 (24)	0/15 (0)		
1BDQ+PMD+MXF+PZA/BDQ+PMD						9/15 (60)	1/15 (7)	0/15 (0)	0/15 (0)
1BDQ+PMD+MXF+PZA/BDQ+PMD+MXF						5/15 (33)	1/15 (7)	0/15 (0)	
BDQ+PMD+PZA		2.52 ± 0.19				12/15 (80)	2/15 (13)		
BDQ+PMD+MXF		4.62 ± 0.27	1.17 ± 0.20	0.16 ± 0.22			15/15 (100)	1/15 (7)	0/15 (0)

a For regimens with two phases, the phases are separated by a slash and the number at the beginning indicates the duration of the initial phase in months.

### Relapse after treatment completion in experiment 2.

Mice receiving the 4-drug combination of BDQ+PMD+MXF+PZA showed rapid sterilization, with the proportions of relapsing mice decreasing from 100% after 1 month of treatment to 24% after 1.5 months and 0% after 2 months (Table 2). Comparison with results in the BDQ+PMD+MXF arm showed that inclusion of PZA in the regimen decreased the duration of treatment by 1 month. However, PZA treatment could be discontinued after the first month without significantly affecting the relapse rate. MXF itself had a smaller but measurable effect on relapse prevention, as evidenced by higher rates of relapse in the BDQ+PMD+PZA arm after 1.5 months of treatment. Moreover, removing both MXF and PZA after the first month had a greater effect on relapses after 1.5 months of treatment than did removing just PZA. BDQ+PMD alone was at least as effective as RIF+INH+PZA. Fig. S2 shows the decreases in the numbers of relapsing mice and the reductions in CFU burdens over time in the different treatment groups. None of the isolates from mice relapsing after 2 or 4 months of treatment harbored PMD-resistant CFU in a proportion greater than that observed for the parent strain. PZA-containing regimens were more effective than BDQ+PMD+MXF alone in reducing the proportions of PMD-resistant CFU among relapsing isolates (*P* < 0.05). The isolate from the 1 mouse that relapsed after receiving BDQ+PMD for 4 months did not grow on BDQ-containing plates.

### Lung CFU counts during treatment and relapse after treatment completion in experiment 3.

This experiment explored the effect of PZA treatment duration on the efficacy of the BDQ+PMD+PZA combination, using PZA treatment durations of 0, 1, 2, and 3 months. RIF+INH+PZA and BDQ+PMD again had similar activities, with BDQ+PMD resulting in slightly fewer relapses after 4 months of treatment (Table 3). The addition of PZA to BDQ+PMD significantly decreased the CFU counts. In the absence of MXF in this experiment, PZA contributed bactericidal and sterilizing activities over the first 2 months, as evidenced by lower CFU counts and fewer relapses after 2 months of treatment when PZA was administered for 2 months rather than 1 month. Only 1 mouse in each of the groups that received at least 1 month of BDQ+PMD+PZA treatment relapsed after 3 months of treatment, and each had a very low CFU count (Fig. S3). Thus, the addition of PZA to BDQ+PMD for 1 or 2 months decreased the treatment duration necessary to prevent relapse by roughly 1 or 2 months, respectively. Because the rate of relapse after 2 months of BDQ+PMD+PZA treatment was already very low, the benefit of continuing PZA treatment for a third month could not be measured. None of the isolates from mice that relapsed after 3 months of treatment harbored an increased proportion of PMD-resistant CFU, compared to the parent strain.

**TABLE 3:** Lung CFU counts assessed during treatment and proportions of mice relapsing after treatment completion in experiment 3

Drug regimen^*a*^	Log_10_ CFU count (mean ± SD)			No. relapsing/total no. (%)			
	Day 0	Month 1	Month 2	Month 1.5	Month 2	Month 3	Month 4
None	8.04 ± 0.15						
RIF+INH+PZA		5.39 ± 0.19	3.12 ± 0.12			15/15 (100)	4/15 (27)
BDQ+PMD		5.32 ± 0.12	3.39 ± 0.14			14/15 (93)	0/15 (0)
BDQ+PMD+PZA						1/15 (7)	
2BDQ+PMD+PZA/BDQ+PMD			0.18 ± 0.25	10/15 (67)	2/15 (13)	1/15 (7)	0/15 (0)
1BDQ+PMD+PZA/BDQ+PMD		2.32 ± 0.36	0.96 ± 0.55	14/15 (93)	10/15 (67)	1/15 (7)	0/15 (0)

a For regimens with two phases, the phases are separated by a slash and the number at the beginning indicates the duration of the initial phase in months.

## DISCUSSION

The experiments presented here demonstrate the substantial sterilizing activity of the novel 4-drug regimen of BDQ+PMD+MXF+PZA in mice. Compared to the current first-line regimen of RIF+INH+PZA, this regimen decreased the duration of treatment needed to prevent similar numbers of relapses by 2.5 to 3.5 months. For example, in experiments 1 and 2 combined, 1.5 months of BDQ+PMD+MXF+PZA treatment cured 78% of treated mice (25 of 32 mice), whereas RIF+INH+PZA treatment cured 50% of treated mice (15 of 30 mice) after 4 months and 87% (13 of 15 mice) after 5 months. This treatment-shortening effect is significantly greater than that observed previously in mice with the substitution of MXF into the first-line regimen, which suggests that the BDQ+PMD+MXF+PZA regimen has greater treatment-shortening potential than the regimens recently studied in the phase 3 REMox-TB trial (10, 11). Inclusion of two novel drug classes also would significantly expand the spectrum of activity of the regimen to include MDR-TB isolates with PZA or MXF resistance. The initial efficacy of this 4-drug combination administered for 8 weeks is currently being studied in MDR-TB patients, with or without PZA-resistant isolates, in the phase 2b NC-005 trial. If the combination is sufficiently efficacious, safe, and well tolerated, then the present results suggest that it may be a more effective regimen for DS-TB than the other BDQ- or PMD-containing regimens currently in clinical trials.

Removal of BDQ, PZA, or MXF reduced the activity of the 4-drug regimen, which confirms the important contributions of these components to the regimen. Removal of BDQ (i.e., administering PMD+MXF+PZA alone) caused the greatest reduction in efficacy (experiment 1), increasing the treatment duration needed to achieve 80% cure (i.e., 20% relapse) by 2.5 months, compared to BDQ+PMD+MXF+PZA. This result suggests that adding BDQ to the PMD+MXF+PZA regimen that is currently under evaluation in the phase 3 STAND trial would significantly increase the regimen's potential to shorten the duration of treatment. The use of BDQ in this 4-drug combination also appears to significantly restrict the selection of PMD-resistant mutants, which was evident in some mice receiving PMD+MXF+PZA alone and occasionally resulted in relapse if the treatment duration was too short (experiment 1). Given the relatively high frequency of spontaneous PMD-resistant mutants, BDQ may have an important role in preventing the selection of nitroimidazole-resistant mutants with novel regimens.

Although not as great as the contribution of BDQ, the contribution of PZA to the BDQ+PMD+MXF+PZA regimen was substantial. Removal of PZA increased the treatment duration required to prevent nearly all relapses by as much as 1.5 months in experiments 1 and 2. Nevertheless, the BDQ+PMD+MXF combination remained superior to RIF+INH+PZA, which suggests that the BDQ+PMD+MXF+PZA regimen would retain strong sterilizing activity even against MDR-TB isolates that were no longer susceptible to PZA (or the innately PZA-resistant Mycobacterium bovis). This is important, given the challenges currently preventing rapid detection of PZA resistance. Since administration of PZA for the full duration of the current first-line regimen is not required, the effect of shortening the duration of PZA treatment in the BDQ+PMD+MXF+PZA regimen was explored in experiment 2. This experiment revealed that continuation of PZA treatment beyond the first month was unnecessary. This result was similar to the recent observation that linezolid (LZD) lends sterilizing activity to the BDQ+PMD combination but is not required for the entire duration (12). To the best of our knowledge, PZA and LZD represent the only drugs for which such a time-limited contribution to the sterilizing activity of a regimen has been observed in mice. Because PZA exerts sterilizing effects for longer treatment periods in other drug regimens, the duration of PZA's contribution to a regimen appears to depend on the sterilizing potential of its companion agents (13). Because intolerance of PZA side effects is not uncommon and fatal hepatotoxicity is occasionally observed, the optimal duration of PZA administration in the BDQ+PMD+MXF+PZA regimen should be explored in future clinical trials.

The contribution of MXF to the efficacy of the BDQ+PMD+MXF+PZA regimen was not as great as that of BDQ or PZA. However, removal of MXF did significantly increase the proportions of mice relapsing after 1.5 months of treatment in experiments 1 and 2. Moreover, continuation of MXF administration beyond the first month enabled discontinuation of PZA administration after 1 month without a significant increase in the relapse rate after 1.5 months of treatment in experiment 2. The BDQ+PMD+PZA regimen currently being studied in the phase 2b NC-005 trial was again superior to the RIF+INH+PZA regimen in mice, as demonstrated previously (8), which suggests that the BDQ+PMD+MXF+PZA regimen would retain strong sterilizing activity in the event of MXF resistance.

Even the 2-drug core of BDQ+PMD alone appears to have sterilizing activity at least comparable to that of the current first-line regimen. However, 3 or more drugs are always recommended for the treatment of active TB. Previous results in mice suggested that, in cases in which MXF and/or PZA use is lost to resistance, the addition of LZD could boost the sterilizing activity of BDQ+PMD±PZA (12). Emerging data from the same model indicate that LZD also increases the bactericidal and sterilizing activities of BDQ+PMD+MXF (data not shown). These data suggest the possibility of treatment algorithms in which a 4-month BDQ+PMD+MXF+PZA regimen is used for DS-TB and the composition and/or duration of the regimen is modified (e.g., by substituting LZD for MXF and/or PZA) based on rapid susceptibility test results. Such an algorithm, if supported by the results of the ongoing STAND, NC-005, and NiX-TB (ClinicalTrials registration no. NCT02333799) trials and future trials, would provide a modifiable treatment regimen that largely meets the target product profile for a “pan-TB” regimen, as recently set forth by the World Health Organization (14).

This study has several limitations. The general limitations related to the use of this murine model to study novel regimens were articulated previously (15, 16). One specific limitation of the experiments presented here is that they did not isolate the contribution of PMD to the BDQ+PMD+MXF+PZA regimen. Experiments to evaluate the role of PMD in a variety of murine models are under way and will be reported separately.

## MATERIALS AND METHODS

### Mycobacterial strain.

Mycobacterium tuberculosis H37Rv was passaged in mice, frozen in aliquots, and subcultured in Middlebrook 7H9 broth with 10% oleic acid-albumin-dextrose-catalase (OADC) (Fisher, Pittsburgh, PA) and 0.05% Tween 80 prior to infection.

### Antimicrobials.

INH, RIF, PZA, BDQ, MXF, and PMD were obtained and formulated for oral administration as described previously (8, 17, 18).

### Aerosol infection with M. tuberculosis.

All animal procedures were approved by the Johns Hopkins University Animal Care and Use Committee. High-dose aerosol infection was performed as described previously (5). Briefly, 5- to 6-week-old female BALB/c mice (Charles River, Wilmington, MA) were infected with Mycobacterium tuberculosis H37Rv using an inhalation exposure system (Glas-Col, Terre Haute, IN) and a fresh log-phase broth culture (optical density at 600 nm of 0.8 to 1.0), with the goal of implanting 3.5 to 4.0 log_10_ CFU in the lungs of each mouse. Two or three mice from each aerosol infection run (experiments 1, 2, and 3) were humanely killed 1 day after infection and on the day of treatment initiation (day 0), to determine the numbers of bacteria implanted in the lungs and at the start of treatment, respectively.

### Chemotherapy.

Mice were block-randomized by aerosol run to experimental arms prior to treatment. Treatment was initiated 14 days after infection. Treatment was administered once daily, by gavage, 5 days per week. Drug doses were 10 mg/kg INH, 10 mg/kg RIF, 150 mg/kg PZA, 25 mg/kg BDQ, 50 or 100 mg/kg PMD, and 100 mg/kg MXF (8, 15, 19). Each drug was administered once daily, unless otherwise indicated. INH and PZA were administered >1 h after RIF. BDQ and PMD were administered sequentially in the morning, followed by MXF and/or PZA, in a single gavage, in the afternoon. Control mice received RIF+INH+PZA for 2 months followed by RIF+INH alone for a total of up to 5 months.

### Assessment of treatment efficacy.

Efficacy was assessed on the basis of lung CFU counts at selected time points during treatment (a measure of bactericidal activity) and the proportion of mice with culture-positive relapse after treatment completion (a measure of sterilizing activity). Quantitative cultures of lung homogenates were performed in parallel on 7H11 agar enriched with OADC (basic agar) and on basic agar supplemented with 0.4% activated charcoal to reduce drug carryover effects (8). Plates were incubated for up to 42 days at 37°C before final CFU counts were determined. Lung CFU counts were assessed in 4 or 5 mice per treatment group at each time point. The proportions of mice with culture-positive relapse were determined by maintaining cohorts of 15 mice for 3 additional months after the completion of treatment and then sacrificing them to determine the proportions with positive lung cultures, defined as 1 CFU of M. tuberculosis detected after plating of the entire lung homogenate onto five 7H11 plates, at least two of which were supplemented with 0.4% activated charcoal.

### Evaluation of resistance selection.

The proportions of CFU able to grow with PMD (2 μg/ml, or approximately 30 times the MIC) and BDQ (0.06 and 0.125 μg/ml, or 2 and 4 times the MIC) were determined at day 0, month 1, month 2, and month 3 and at relapse time points by plating serial dilutions of the lung homogenates directly on basic 7H11 agar containing the indicated drug concentrations.

### Statistical analysis.

CFU counts (*x*) were log-transformed (as *x* + 1) before analysis, and group means were compared by one-way analysis of variance, with Dunnett's posttest to control for multiple comparisons. Group relapse proportions were compared using Fisher's exact test, adjusting for multiple comparisons. The Mann-Whitney test was used to test for significance for non-normally distributed relapse CFU data. GraphPad Prism 6 (GraphPad, San Diego, CA) was used for all analyses. The use of 15 mice per group for relapse assessment provided approximately 80% power to detect 40-percentage-point differences in the relapse rate, with α set at 0.01 to adjust for up to 5 simultaneous two-sided comparisons. Smaller differences may not be meaningful in terms of shortening the duration of treatment.

## Supplementary Material

Supplemental material
