# Supplementary material for: Bactericidal and Sterilizing Activity of a Novel Regimen with Bedaquiline, Pretomanid, Moxifloxacin, and Pyrazinamide in a Murine Model of Tuberculosis
Source: Antimicrob Agents Chemother. 2017 Aug 24;61(9):e00913-17. doi: 10.1128/AAC.00913-17 (PMC5571308; doi:10.1128/AAC.00913-17)
Supplement: Supplemental material [file supp_61_9_e00913-17__index.html]

Supplemental material 

# Bactericidal and Sterilizing Activity of a Novel Regimen with Bedaquiline, Pretomanid, Moxifloxacin, and Pyrazinamide in a Murine Model of Tuberculosis

## Supplemental material

- Supplemental file 1 -

  Supplemental Figures S1 to S3

  PDF, 92K
