# Supplementary material for: Bactericidal and Sterilizing Activity of a Novel Regimen with Bedaquiline, Pretomanid, Moxifloxacin, and Pyrazinamide in a Murine Model of Tuberculosis
Source: Antimicrob Agents Chemother. 2017 Aug 24;61(9):e00913-17. doi: 10.1128/AAC.00913-17 (PMC5571308; doi:10.1128/AAC.00913-17)

Supplemental Figure 1. Lung CFU counts at the time of relapse assessment in Experiment 1. Bars show median CFU counts. Mice held for relapse after 1.5 months of treatment with JPaMZ had significantly ($p= 0.0004$) fewer CFU compared to the mice treated with JPaZ only (Mann-Whitney). At M4, the CFU counts in RHZ-treated mice were significantly higher than those in either PaMZ-treated group before, but not after, adjustment for multiple comparisons (Mann-Whitney).

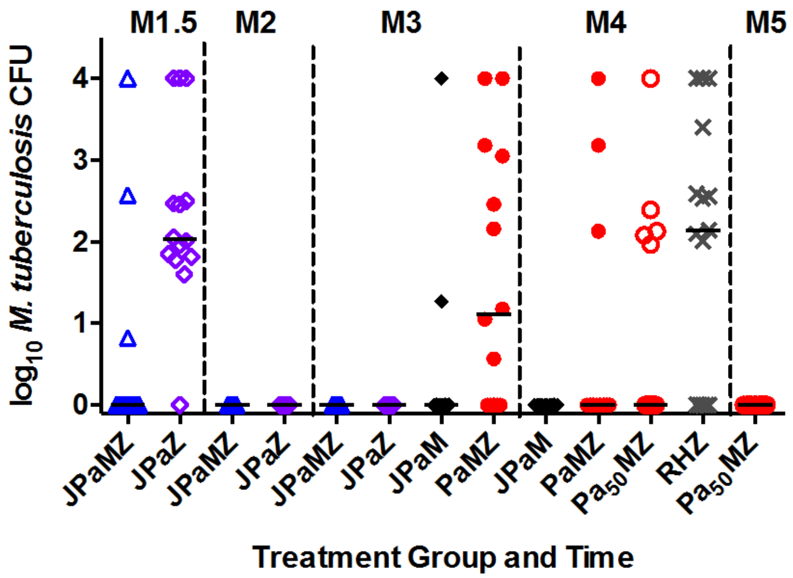

Abbreviations: R=RIF, H=INH, Z=PZA, Pa=PMD, M=MXF, J=BDQ

Supplemental Figure 2. Lung CFU counts at the time of relapse assessment in Experiment 2. Bars show median CFU counts. Compared to mice receiving 1.5 months of JPaMZ, mice that had discontinued PZA or PZA and MXF (i.e., a continuation with JPaM or JPa) after the first month were not significantly different whereas mice that had had no MXF had statistically significantly higher CFU counts ($p=0.0053$, Kruskal-Wallis, Dunn's). After 2 months of treatment, all of the regimens were statistically comparable to JPaMZ except for the mice that had never received PZA ($p<0.0001$, Kruskal-Wallis, Dunn's).

Abbreviations: R=RIF, H=INH, Z=PZA, Pa=PMD, M=MXF, J=BDQ

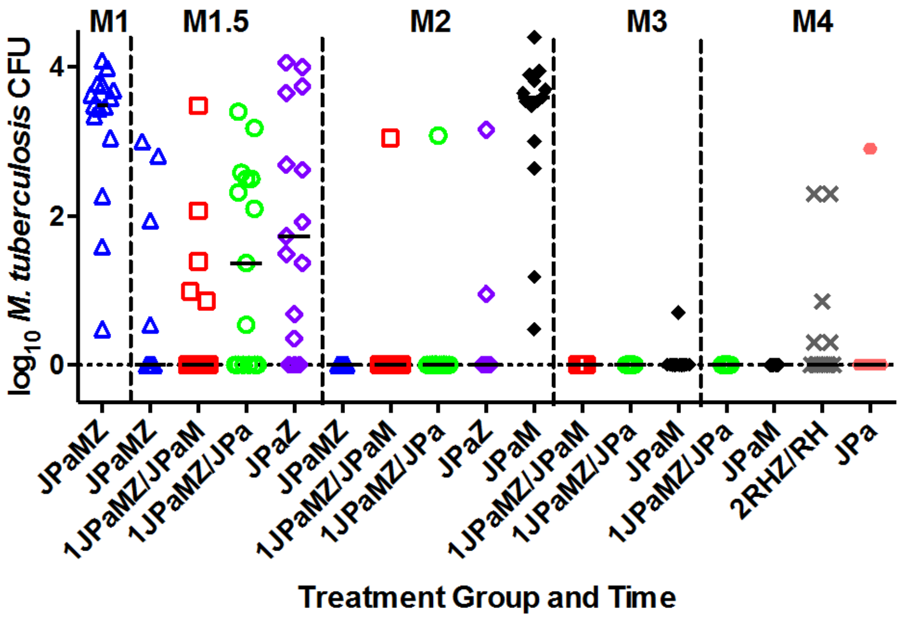

Supplemental Figure 3. Lung CFU counts at the time of relapse assessment in Experiment 3. Bars show median CFU counts. Mice were treated with three months with BDQ+PMD+PZA (open inverted green triangles), with BDQ+PMD+PZA for two months followed by BDQ+PMD (solid blue circles), or with BDQ+PMD+PZA for one month followed by BDQ+PMD (solid light blue triangles). After 1.5 and 2 months of treatment, mice that received PZA beyond the first month had significantly fewer CFU than mice receiving PZA for one month only ($p=0.0438$ and $p=0.0023$, respectively, by paired T test). After 3 months of treatment, there was no statistically significant difference between the JPaZ-containing regimens. However, the JPaZ-containing regimens were superior to JPa and RHZ ($p<0.0001$). The results support the use of PZA for at least two months to achieve more rapid and complete sterilization (i.e. by M2 or M3). Mice treated with BDQ+PMD (red open squares) were all cured after 4 months of treatment but the difference with the RHZ group was not statistically significant.

Abbreviations: R=RIF, H=INH, Z=PZA, Pa=PMD, J=BDQ

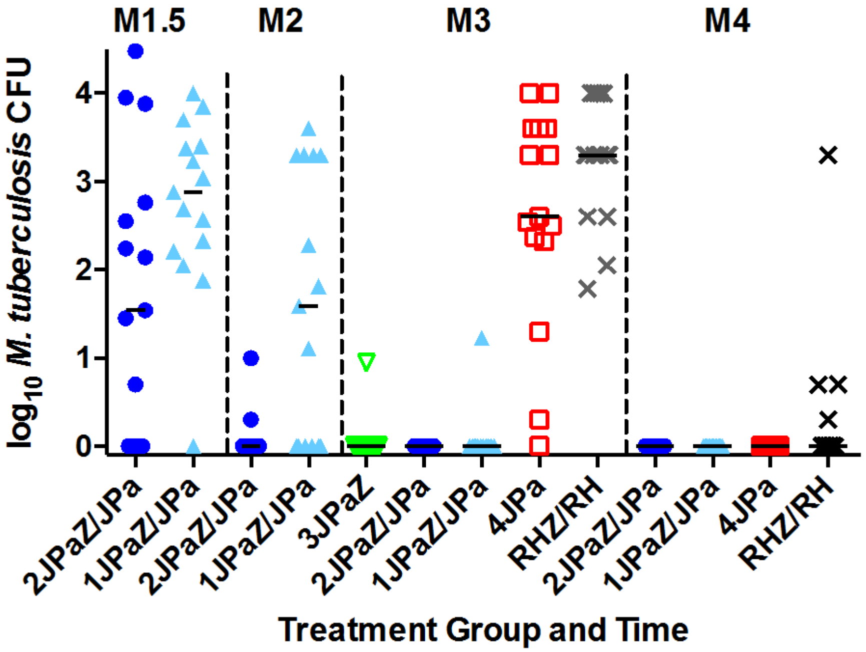

Supplement: Supplemental material [file AAC.00913-17_zac009176476s1.pdf]
